# Signatures of personality on dense 3D facial images

**DOI:** 10.1038/s41598-017-00071-5

**Published:** 2017-03-06

**Authors:** Sile Hu, Jieyi Xiong, Pengcheng Fu, Lu Qiao, Jingze Tan, Li Jin, Kun Tang

**Affiliations:** 1 Key Laboratory of Computational Biology, CAS-MPG Partner Institute for Computational Biology, Shanghai Institutes for Biological Sciences, Chinese Academy of Science, Shanghai, 200031 China; 2 Berlin Institute for Medical Systems Biology, Max-Delbrueck-Center for Molecular Medicine, Berlin, Germany; 3 Department of Neurology, the First People’s Hospital of Chenzhou, Hunan, 423000 China; 4 State Key Laboratory of Genetic Engineering and Ministry of Education Key Laboratory of Contemporary Anthropology, School of Life Sciences, Fudan University, Shanghai, 200433 China

## Abstract

It has long been speculated that cues on the human face exist that allow observers to make reliable judgments of others’ personality traits. However, direct evidence of association between facial shapes and personality is missing from the current literature. This study assessed the personality attributes of 834 Han Chinese volunteers (405 males and 429 females), utilising the five-factor personality model (‘Big Five’), and collected their neutral 3D facial images. Dense anatomical correspondence was established across the 3D facial images in order to allow high-dimensional quantitative analyses of the facial phenotypes. In this paper, we developed a Partial Least Squares (PLS) -based method. We used composite partial least squares component (CPSLC) to test association between the self-tested personality scores and the dense 3D facial image data, then used principal component analysis (PCA) for further validation. Among the five personality factors, agreeableness and conscientiousness in males and extraversion in females were significantly associated with specific facial patterns. The personality-related facial patterns were extracted and their effects were extrapolated on simulated 3D facial models.

## Introduction

As one of the most complex anthropological traits, the human facial shape is strongly regulated by many factors such as genetics, ethnicity, age, gender, and health. Due to the rapid progress of facial imaging and analysis technology, especially the 3D dense face model-based approaches, complex facial shape traits are continuously being discovered in order to signal genetic polymorphisms^1^, ethnicity^2^, gender^3^, diseases^4^, health^5^, as well as aging^6^.

Apart from anthropological perspectives, the human face was also intensively studied for its social attributes; *e.g.* many aspects of the human personality are honestly signaled on the human face^7–9^, and many other socially relevant traits, such as attractiveness^10, 11^, aggression^12^, and sociosexuality^13^ can also be identified on the human face. From an evolutionary perspective, human faces have evolved to signal individual identity^14^ and served adaptive functions to promote genetic fitness^15^. Moreover, faces represent an important signaling system not only in humans, but also in chimpanzees, which accurately pictures the personality, indicating shared signaling structures across two species^7^. Nevertheless, the facial signals of social attributes are more difficult to study due to the involute and highly subjective nature of social attributes. Many people believe that facial cues exist towards the hidden personality of unknown individuals^16^. Indeed, a series of studies have been carried out to formally test the hypothesis of facial cues of personality. Most of these studies utilised the five- factor model of personality, or the ‘Big Five’ (BF) model^17–19^. The BF model ascribes the personality into five dimensions: extraversion (E), agreeableness (A), conscientiousness (C), neuroticism (N), and openness (O). According to the definitions, a higher score of E, A, C, N, and O indicates one’s personality of being outgoing or energetic, friendly or compassionate, self-disciplined or organised, sensitive or nervous, and inventive or curious, respectively. The BF model is well suited for the purpose of evaluating the facial signals of personality due to several advantages. First, the five personality factors were found to be approximately orthogonal to each other^20^, as is the desirable statistical property in factor analyses. Second, people’s BF test scores are highly stable during adulthood^21^. In fact, genetic studies revealed relatively high heritability (42~57%) of the five personality traits^22^, suggesting that the BF traits reflect more constitutional characteristics rather than transient emotional changes. Finally, the BF model has been successfully applied to different genders^23^, a variety of cultures^24, 25^, and even in chimpanzees^26^, showing its strong cross-group robustness and compatibility. Strong evidence in previous studies also verified that BF scores correlated with a number of behaviors, and sometimes even play a determinant role in behavior prediction, demonstrating tremendous potential applications for the BF theory^27–29^.

Passini and Norman first conducted a seminal study in 1966, in which a small group of volunteers, unknown to each other, were asked to rate themselves and their peers on the BF scales without verbal communication^30^. They found that for extraversion, agreeableness, and openness, the self-reported scores and those scored by observers matched significantly. Other similar studies also confirmed that people can correctly recognise the personality of unknown people to certain extents, during the first encounter, without the aid of verbal communication^16, 31^. Later studies noted that the cues for personalities could be recovered in mere static face pictures with neutral expressions^9, 32, 33^. In order to directly obtain the specific facial patterns associated with personality traits, Little and Perrett proposed an ingenious method^33^. They ranked the head portraits of the volunteers along the five BF dimensions based on their self-ratings, and synthesised the composite portraits for the extreme scorers. They found that raters could identify the BF traits underlying the composite images better than chance, particularly for conscientiousness and extraversion and agreeableness. Similar studies confirmed the accuracy of composite images in guiding the recognition of personality traits^5, 9^.

Nonetheless, all of the previous studies relied on the subjective judgment of human raters to evaluate the effects of particular facial signals; a direct association between facial shape changes and the personality is missing. In other words, we still do not know whether the inner personality induces substantial modulations on an individual’s physical facial appearance; and even if it does, what are the exact physical changes pertaining to each personality attribute? This question is particularly important for the feasibility of automatic facial recognition systems that may judge personalities without human involvement. Furthermore, all the previous studies were conducted in relatively small samples, and their photo collection solely relied on the 2D systems, which easily resulted in artificial explanations by introducing too many confounding, but inevitable factors, such as view angle and posture difference^34^. Fortunately, the 3D scanning technology successfully overcame such drawbacks, *e.g.* the problem of the view angle can be addressed through taking photos over different angles simultaneously by a 3D camera. Moreover, the 3D scanning can provide both the information of one’s facial shape and the facial surface (texture), thereby facilitating the performance of capturing the essential features from human faces to offer more precise predictions of the correlation between the human face and the human personality^35^. In the current study, we followed a novel strategy by utilizing 3D images to uncover the association between the human face and the corresponding personalities. In brief, we collected 3D expression-neutral facial images of 834 volunteers from Shanghai, China, using the high-resolution 3D camera system (3dMD Face System, www.3dmd.com), as well as their corresponding BF questionnaires, in which the results can be transferred to the consecutive score for each BF factor. Thereafter, we proposed a partial least squares (PLS) based statistical method called CPLSC to inspect the association between the human face and each of the BF factors based on the 3D image data and the BF scores, and extract the personality-related facial features from the high-density 3D image data. In addition, we further validated the results from CPLSC by PCA (principal component analysis). All the extracted personality-related features were finally visualised and animated by our R package “3DFace”.

## Results

### Personality test using BF model

Personality scores were measured for all the volunteers using a self-report questionnaire (Chinese Version) according to the BF Inventory. Cronbach’s α coefficient was used to evaluate the reliability of the BF scores^36^. Cronbach’s α reflects the correlation of different tests towards the same personality factor that is to be measured. In our survey, the Cronbach’s α scores ranged from 0.68 to 0.80 with a mean of 0.74 (Table 1), consistent with the previous surveys in East Asians^37^.

**Table 1:** Cronbach’s α in each of BF factor in two genders.

Personality	Male	Female
Extraversion	0.80	0.79
Agreeableness	0.71	0.68
Conscientiousness	0.72	0.73
Neuroticism	0.70	0.71
Openness	0.76	0.77

### Association analyses of 3D facial images and personality based on PLS regression

**Figure 1:**
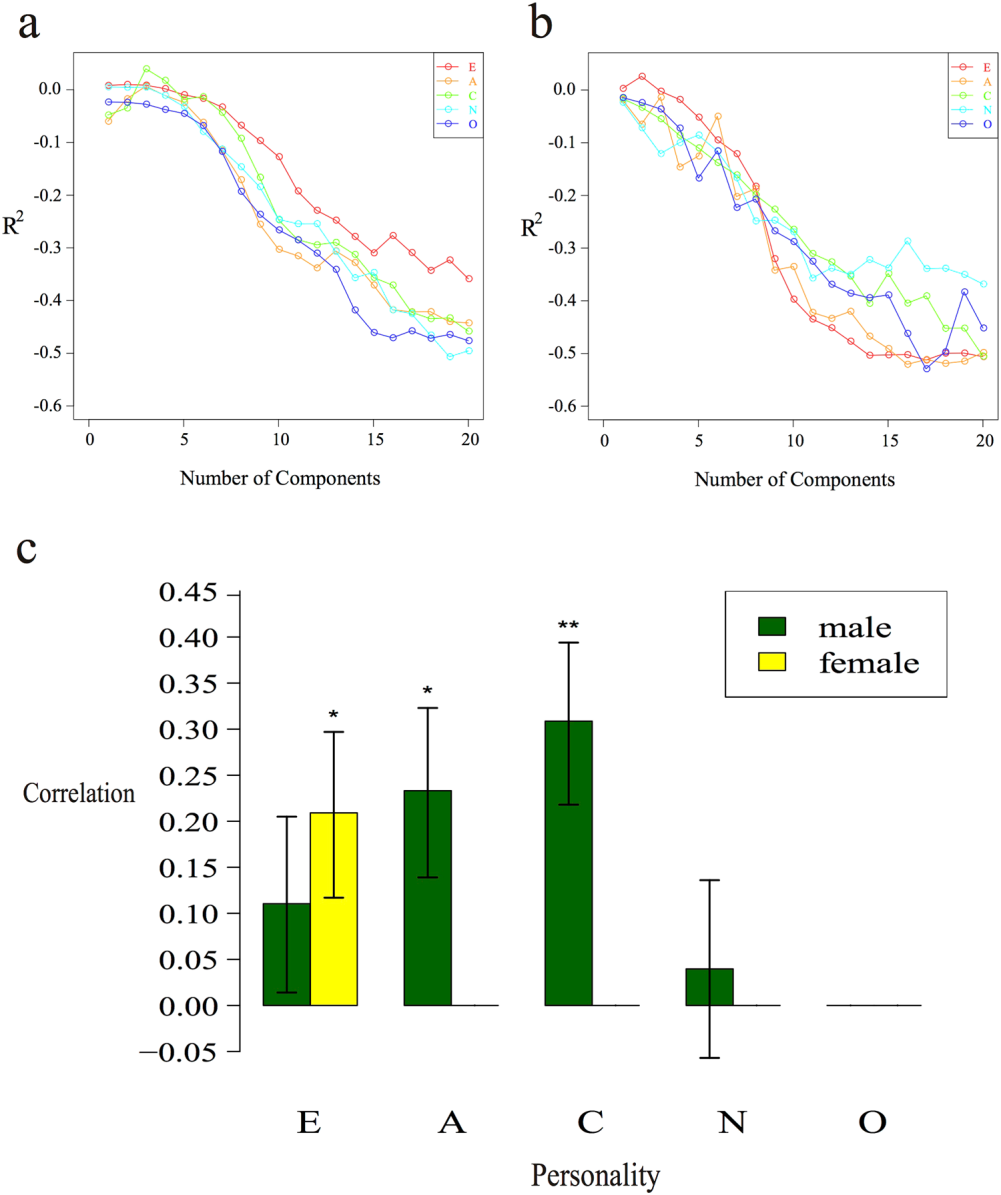
Determining the effective number of PLSCs for each BF factor, and personality traits significantly correlated between predicted and true scores. (**a**) R^2^ evaluation to determine the effective number of PLSCs number in male samples. (**b**) R^2^ evaluation to determine the effective number of PLSCs in female samples. (**c**) Personality traits with significant correlation between the predicted and the true scores.

PLS is a statistical approach to find the maximum potential associations between two variables, either or both of which could be multi-dimensional. It is, therefore, well suited for finding the latent relationships between the BF factors and the high-dimensional facial morphological data. To control the gender effect, we conducted the analyses in males and females separately throughout the study. Within each gender group, we carried out PLS regressions for each of the five BF factors separately, and a leave-one-out (LOO) procedure was applied to cross-validate the PLS models (see methods). Similar to the PCA, PLS can also iteratively decompose the high-dimensional data space into consecutive PLS components (PLSC)^38^. An optimal PLS model is the one composed of the top-ranking PLSCs that can best predict independent data. For each BF factor, we analysed up to top 20 PLSCs, and the coefficient of determination (*R* ^*2*^) was calculated on the cross-validation data to evaluate the accuracy of prediction (Fig. 1a,b, Method). Positive *R* ^*2*^ values indicate effective predictions, and greater *R* ^*2*^ values stand for better performance; on the contrary, an *R* ^*2*^ curve, which is constantly below zero, indicates lack of association signals, and thus no predictive power towards the corresponding personality trait. We define the effective PLSC number as the number of top PLSCs that rendered the maximum positive *R* ^*2*^ value. As can be seen in Fig. 1a and b, the effective PLSC numbers are 2, 3, 3, 2 for E, A, C and N in males, and 2 for E in females, respectively. For the other traits, *R* ^*2*^ values were always negative and they were dropped from further PLS analyses.

For the personality traits that can be effectively predicted, we obtained the Pearson correlation ρ between the true BF scores and the corresponding predicted scores from the LOO procedure, under the optimal prediction models. In order to formally test the statistical significance, these correlation scores were compared to null distributions generated by re-shuffling the BF scores among different individuals (see Methods for details). As shown in Fig. 1c, we found that the 3D facial shapes were significantly associated with personality trait A (Pearson’s correlation ρ = 0.233, permutation *p value* = 0.032) and C (Pearson’s correlation ρ = 0.309, permutation *p value* = 0.008) in males, and E (Pearson’s correlation ρ = 0.209, permutation *p value* = 0.035) in females. For the personality trait E (Pearson’s correlation ρ = 0.111, permutation *p value* = 0.147) and N (Pearson’s correlation ρ = 0.04, permutation *p value* = 0.334) in males, although the correlations were positive, the reshuffling test did not support their statistical significance, suggesting that such moderate positive correlations may appear merely by chance.

**Figure 2:**
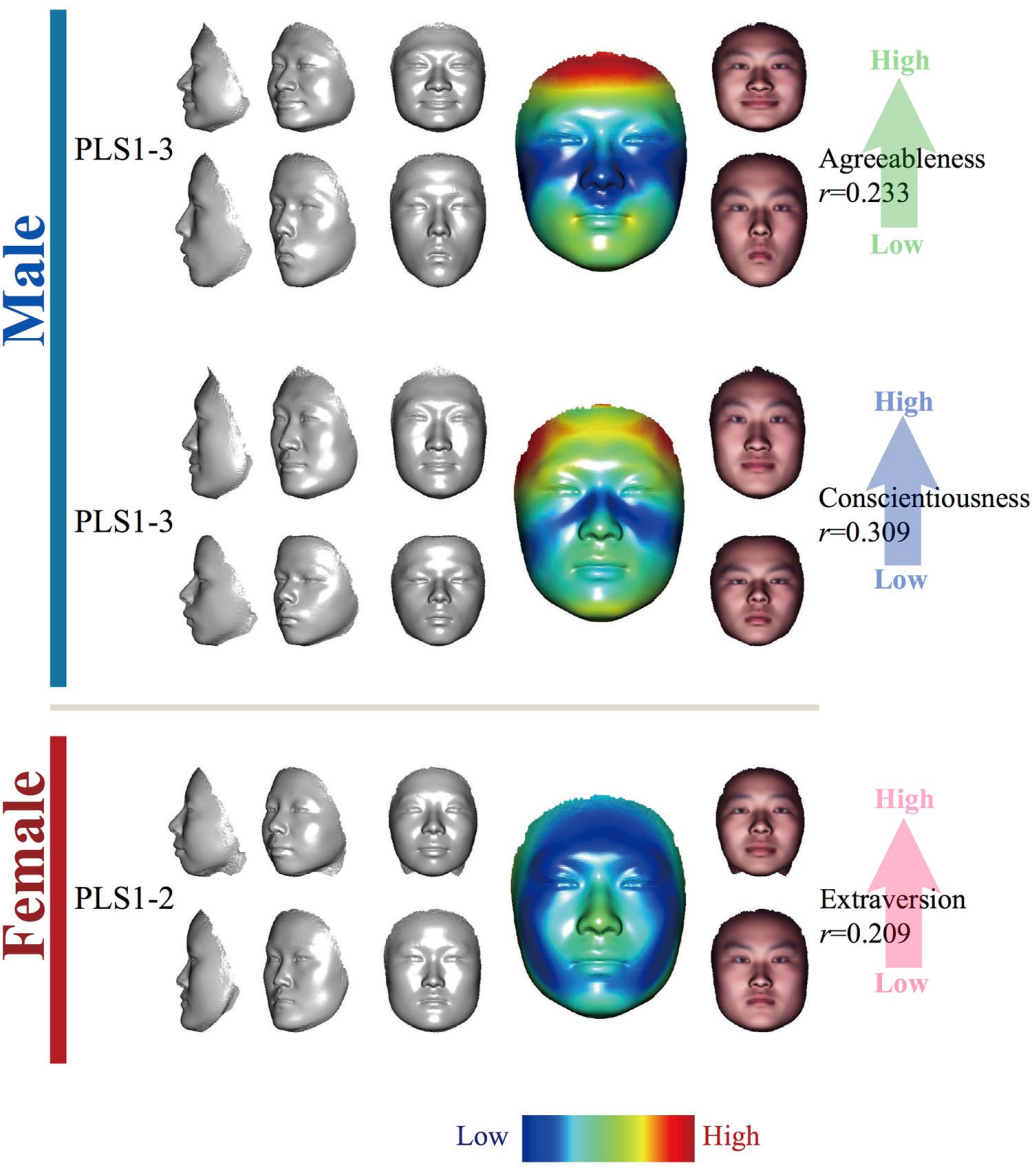
Features selected by CPLSC model from faces significantly associated with BF factors in two gender. Three panels are sequentially ordered from top to bottom: agreeableness-related CPLSC, which consists of the first three PLSCs in males; conscientiousness-related CPLSCs, which consists of the first three PLSCs in male; extraversion-related CPLSC, which consists of the first two PLSCs in female. In each panel, the upper faces are simulated by adding five standard deviations of the projected samples to the mean face; the lower faces are created by subtracting the standard deviation of the projected samples to the mean face. From left to right are faces rotated by 90°, 45°, and 0°. The bigger face following next is the mean face, on which the heat colors represent the norm value of CPLSC at each vertex. At the right side of the mean face are two faces the same as the faces at the left side of the mean face, but with a texture generated from a sample mean face.

For the personality traits showing significant correlations with facial shapes, it is desirable to extract the personality-related facial signatures, as well as to visualise the association that the personality traits induce on the human face. To this effect, we designed the CPLSC method to extract the personality-related facial signatures from the optimal PLS prediction models (see Methods for details). CPLSC basically identifies a single dimension in the 3D face data space that defines the overall shape changes along with an increasing personality score. As a consequence, we extracted A and C related CPLSCs from male samples and E related CPLSCs from female samples. Considering that both personality and face shape vary with age^39^ (Supplementary Table 1), age could be a factor influencing the correlation between facial shape and personality. Therefore, we also calculated the correlations between these personality-associated CPLSCs and age to figure out whether these associations of personality-facial features were attributed by age (Supplementary Table 2). The result shows that all such correlations are not statistically significant, indicating the features we extracted are unrelated with age. The effects of such facial signatures may be clearly visualised by extrapolating the mean face to extreme extents in opposite directions along the CPLSC dimension (Fig. 2; See Methods for details). The results of A and C in males, and E in females are shown in Fig. 2. The heat colour portraits indicate the contribution of each vertex to the shape changes (Methods), with warmer colours signaling greater changes along the CPLSC dimension, and colder colours for minor changes. For agreeableness in males, as shown in the top panel of Fig. 2, the CPLSC signature mostly involved an upper facial region around the forehead and the eyebrows and a lower facial region centred on the lower lip. When we gradually changed the mean face along the CPLSC dimension towards higher agreeableness (see Fig. 2; Supplementary Video), the eyebrows seem to be lifted up with a reduced forehead span (distance between the eyebrows and the hairline). A more recognisable expression appears around the mouth where the lips laterally extend outwards and bend upwards at the lip corners, showing a clear expression of a “smile”. When the face is morphed towards lower agreeableness, opposite changes happen, including sunken eyebrows and jaw; the lip corners also drop downwards to symbolise unhappiness. As for conscientiousness in males, the face of the higher C score shows lifted and laterally extended eyebrows, as well as wider opened eyes. Changes in the lower facial area mainly involve a withdrawn upper lip and pressed muscles around the jaw, demonstrating a pose of tension around the mouth area. These are in contrast to the seemingly relaxed face associated with a low C score, whose eyebrows and eyes are naturally pulled down by gravity, and the muscles around the mouth seem to be rather relaxed. It is interesting to note that the faces of the low A and the low C scores are similar in the general trend: both faces showed a sense of relaxation and indifference.

The only personality trait found significantly associated in females is extraversion. As shown in Fig. 2, the CPLSC model indicates that substantial signals come from around the nose and the upper lip. The regions defining the face contour, including the temples, masseters, and chin also seem to contribute to the shape differences. When morphed along the CPLSC dimension, the face of the higher E score shows a more protruding nose and lips, and recessive chin and masseter muscles. The face of the lower E score clearly shows opposite changes, whose naso-maxillary region seems to press against the facial plane.

### Association analyses of 3D facial images and personality based on PCA

**Figure 3:**
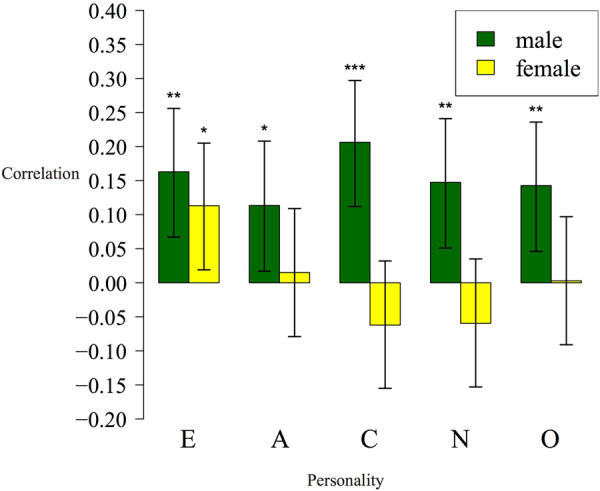
Pearson correlations between surveyed personality scores and the predicted ones under the LOO procedure. A linear model is used to predict each personality score based on 20PC facial data. p-values and significances of correlation tests are labeled above the bar as numbers and asterisks respectively. *p <= 0.05; **p <= 0.01; ***p <= 0.001.

To further validate our result, we used principal component analysis (PCA) to test the potential associations between high-dimensional facial data to the personality traits. The facial data were first decomposed by PCA. Only the top 20 PCs were used in the subsequent analyses, which composed the majority (96.7% in males and 96.1% in females) of the shape variance. Linear regression was used to find the prediction model of personality based on all 20 face PCs, and a similar LOO procedure was used to cross-validate the association (see Methods). Applying the Pearson correlation test between the real personality scores and the predicted ones, all five personalities in males manifested significant predictions: E (Pearson’s correlation ρ = 0.163, *p value* = 0.001), A (Pearson’s correlation ρ = 0.114, *p value* = 0.022), C (Pearson’s correlation ρ = 0.206, *p value* = 2.87 × 10^−5^), N (Pearson’s correlation ρ = 0.148, *p value* = 0.003), and O (Pearson’s correlation ρ = 0.143, *p value* = 0.004), respectively. Meanwhile, the personality E (Pearson’s correlation ρ = 0.113, *p value* = 0.019) in females was significantly better than random (Fig. 3). In general, PCA revealed similar association patterns compared to that of CPLSC: males exhibited tentative or significant signals in most personal traits; conscientiousness in males could be predicted well, and females showed substantial correlation only for extraversion. Differences also exist: associations of E, N and O are strongly significant in PCA, but are weak or missing in the CPLSC analysis.

**Figure 4:**
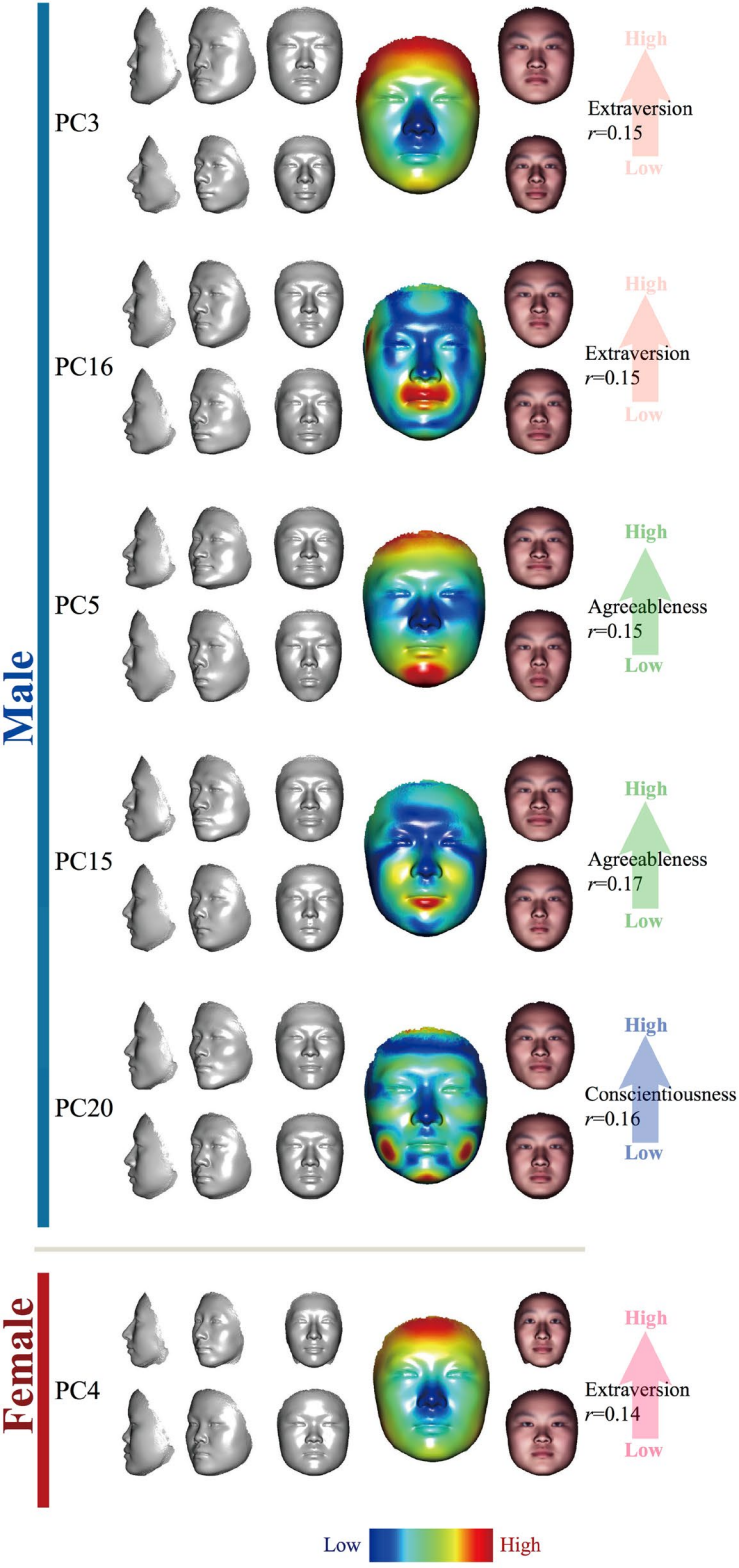
Extracted personality-related feature, which has a significant correlation between predicted score and true score by PCA. From top to bottom are extraversion-related PC3 and PC16 in males, agreeableness-related PC5 and PC15 in males, conscientiousness-related PC 20 in males, extraversion-related PC14 in females. For each panel, the upper faces are created by adding five standard deviations of the projected samples to the mean face; the lower faces are created by subtracting the standard deviation of the projected samples to the mean face. From left to right are faces rotated by 90°, 45°, and 0°. The bigger face next is the mean face, on which the heat colours represent the norm value of PCA at each vertex. At the right side of the mean face are two faces the same as the faces at the left side of the mean face, but with a texture generated from the sample mean face.

Next, we sought to find out the specific facial features that correlated with personality. We calculated the Pearson correlation in the all of the 200 combinations of 20 PCs, 5 personalities, and 2 genders. In order to estimate the false discovery rate, we randomly shuffled the sample labels and re-calculated the Pearson correlation 10000 times (see Methods). The cutoff of the correlation p-value thus defined was 0.0033 for the lowest FDR. In total, six PCs were found to be significantly associated with personality, including PC3 and PC16, which correlated with extraversion, PC5 and PC15, which correlated with agreeableness, and PC20, which correlated with conscientiousness in males; in females, PC4 is correlated with higher extraversion (Fig. 4). Moreover, only average 0.676 false-positives were found under permutation (FDR = 0.11). It is worth noting that for the shared association signals, CPLSC and PC exhibited similar facial variations. In males, the CPLSC model for agreeableness revealed a very similar pattern as PC5, which also showed the strongest association to the A score (Figs 2 and 4). For conscientiousness in males, the CPLSC and the PC20 faces with higher C scores similarly showed tensed jaw muscles (Figs 2 and 4). In females, the PC4 that correlates strongly with E scores showed high similarity to the CPLSC model of extraversion (Figs 2 and 4). Additionally, we also checked whether these six personality-associated facial features are correlated with age (Supplementary Table 2). Only PC5 and PC16 found significant Pearson correlation with age in males (PC5: r = −0.174, *p value* = 4.38 × 10^−4^; PC16: r = 0.101, *p value* = 0.041). After removing the controlling variable of age, we found that the partial correlation between PC5 and agreeableness (r = −0.15, *p value* = 0.003), and the partial correlation between PC16 and extraversion (r = −0.138, *p value* = 0.005), are still significant. Therefore, these correlations between PC and personality were not just due to age.

## Discussion

Reading an individual’s personality from one’s face is a fascinating issue. Previous studies based on static 2D images show that facial traces allow observers to judge personality with certain accuracy, but concrete signatures of morphological changes were not obtained. This is mainly due to the difficulty of extracting morphological features from 2D images. With the development of 3D imaging technology and related analytic methods, it is now possible to identify personality-related facial features through high density 3D facial image data.

In this study, we developed an integrative analysis pipeline, CPLSC, to study the association between 3D faces and personality factors, and extract effective personality-related features from the image data. Due to the complexity of the human face, the variances of personality scores which can be explained by CPLSC is usually small in terms of R^2^ measurement (0.67~3.95%). However, we submit that this study provided the first quantitative morphological signatures associated with personality factors and explained some personality-related variances from the human face by extracting the facial features highly correlated with personality traits and visualising them. Future studies aiming to “read” personality and other psychometric traits based on 3D facial images can benefit from this study.

We used CPLSC to carry out the analysis and PCA for further validation. In general, there is a clear overlap in the signals identified by both methods. Agreeableness and conscientiousness in males, and extraversion in females showed statistical significance and resembled facial features in both CPLSC and PCA analyses. The CPLSC method is based on PLS. PLS is a latent variable approach that is supervised to find the maximal fundamental relationships between the independent variables (e.g. the BF factors) and responses (e.g. the face shape). CPLSC summarises the face-personality associations identified by major PLS components, and is likely to represent the exact facial changes induced by personalities. However, the formal statistical testing of PLS signals involves complicated LOO and re-shuffling procedures to avoid artificial effect, which may sacrifice the overall statistical power of the CPLSC. PCA on the other hand is unsupervised and straightforward in the statistical testing of the face/personality associations. As expected, PCA tests largely repeated the finding of CPLSC. The combined analyses of the top 20 PCs further revealed potential association between extraversion and face in males. For the confirmation of the personality-associated features, subtle facial changes due to personality may appear in minor PC components. We dealt with this problem by examining up to 20 single PCs, some of which compose only minor fractions of the total variance. The risk of getting more false positive signals in minor PCs is avoided by introducing stringent reshuffling and FDR procedures. On the other hand, single PC-related facial features should be interpreted with caution. As PCA decomposition is unsupervised, the facial variation pertaining to each PC mode may not be exclusively accounted for by personality, but may be partially associated with a BF factor by chance. It is the reason why we only find age–correlated facial features in PCs but not in CPLSCs. Also, this could explain why some PC faces of the same BF factor seem to change in opposite directions: in males, PC3 and PC16 are both significantly correlated with extraversion/introversion. However, for PC3, the face of higher extraversion scores is wider and rounder, whereas for PC16, the high-extroversion face is associated with a narrower jaw (Fig. 4). The facial signals of extraversion (PC4) in females also seem to reverse the PC3 pattern in males: the thinner face of female PC4 with a pointier nose seems to indicate higher extraversion than the broader face (Fig. 4). These evidences suggest that characteristic features given by PCA may not be very reliable. For all the facial features extracted by the two methods employed, necessary measurements were performed to avoid confounding and artificial effects. One of the most important steps is to control the neutral expression for all the volunteers participating in the experiments (see Methods).

Overall, our results suggest that among the Han Chinese, a male’s personality is more apparent on his face than a female’s is on hers, and this is supported by both CPLSC and PCA methods. This trend is different from previous studies in European populations^9, 33^, suggesting a role of socio-cultural difference in the exhibition of personality. In our study, agreeableness, conscientiousness and extraversion seem to be more recognisable than other personality traits, as is generally consistent with many previous studies^9, 31, 33, 40^. It still would take further detailed studies, especially with much bigger sample sizes, to answer the question of whether and why personality traits can be perceived from facial features in different intensities. After all, given the solid evidences of face/personality associations identified in this study on a purely quantitative basis without using human raters, it can be argued that all the BF factors and any other psychological traits may leave some morphological cues on the face, which can be “read” by an automatic image processing software. To achieve this, a bigger sample size is needed; methods should be designed to focus on facial regions that are sensitive to the targeted personality traits, and temporal dimension may be added to capture subtle facial movements related to personality in 4D data.

## Methods

### Sample

In order to assess their BF personality scores, 405 male and 429 female Han Chinese volunteers from Fudan University, Shanghai, participated in the 3D image collection and answered the questionnaire. All the participants are Chinese residents; the males fall within the age group of 16–35 years (mean = 21.66, SD = 3.69, Supplementary Figure 1), and the females fall within the age group of 15–42 years (mean = 21.65, SD = 3.76, Supplementary Figure 1). A sample collection for this study was carried out with the approval of the ethics committee of the Shanghai Institutes for Biological Sciences (SIBS) and in accordance with the standards of the Declaration of Helsinki. During the sample collection, all the volunteers were asked to remove makeup, glasses, or anything that may influence the neutral face appearance. The volunteers were also asked to maintain a standard posture: looking directly ahead, sitting straight, and pulling back the hair to expose the forehead. In the post-image processing, we also manually checked the 3D images and filtered out photos with low quality and displaying apparent expression before automatic image alignment. The individual texture information was also discarded after the 3D facial alignment. All the methods were carried out in accordance with the corresponding guidelines and regulations. A written statement of informed consent was obtained from every participant, with his/her authorising signature. All facial images in the figures and the supplementary videos of this manuscript were generated by averaging the faces of all the subjects engaged in this study followed by mathematical transformations; the real facial image of any individual participant is not shown in this manuscript.

### 3D Image Acquisition and Processing

All the 3D facial images of the sample were collected by a high-resolution 3D camera system (3dMD face system, www.3dmd.com/3dMDface ); a dense non-rigid registration is then applied to align all the 3D images according to anatomical homology^41^. After the alignment, each 3D facial image data contains 32251 3D vertices.

### The Big Five Inventory

The Big Five Inventory^42^ is downloaded from the Berkeley Personality Lab website (http://www.ocf.berkeley.edu/~johnlab/bfi.htm) and translated into Chinese. This questionnaire is based on the personality model of the five factors, including extraversion (E), agreeableness (A), conscientiousness (C), neuroticism (N) and openness (O). The whole inventory is composed of 44 questions in short and easy-to-understand phrases. Each question is designed to be self-rated in a 1 to 5 scale. Cronbach’s α in psychometrics is used to evaluate the robustness of our survey^36^.

### Composite Partial Least Square Components

In our study, we proposed the CPLSC framework to integrate the features obtained from individual PLSC^43^. Briefly, PLS is a kind of dimension reduction method used when dealing with problems in which the number of factors is much larger than the sample size, or the factors are highly collinear. The basic assumption for PLS is that the data observed is produced by a system driven by a few of the latent variables^44^. We used the R package “pls” developed by Björn-Helge Mevik and Ron Wehrens to carry out the PLS analysis in this study^45, 46^.

The CPLSC method begins with the determination of the effective number of PLSCs. We introduced coefficients of determination R^2^ to evaluate the performance of the model predictions with an increasing number of PLSCs. The definition of R^2^ is as follows: assuming we have a set of data ***u*** = (*u*_1_, *u*_2_, …, *u*_n_), where *u*_*i*,_ *i* = 1, 2, 3…*n* and corresponding prediction ***f*** = (*f*_1_, *f*_2_, …, *f*_n_), where *f*_*i*_, *i* = 1, 2, …, *n*. and is the mean of *u*_*i*_, *i* = 1, 2, 3 … *n*. Then the total sum of square is:

1 $${S}_{tot}=\frac{1}{n}\sum _{i=1}^{n}{({u}_{i}-\bar{u})}^{2}$$

the residual sum of square is:

2 $${S}_{res}=\frac{1}{n}\sum _{i=1}^{n}{({u}_{i}-{f}_{i})}^{2}$$

So, the definition of R^2^ is:

3 $${R}^{2}=1-\frac{{S}_{res}}{{S}_{tot}}$$

The prediction ***f*** is obtained by the LOO procedure. In brief, given *N* individuals in a sample, one individual is left out, and a prediction model is constructed based on the rest *N-*1 individuals using PLS regression. This model can be used to predict the personality *f*_*i*_ of the left-out individual based on its facial data. The LOO procedure is repeated for every individual. The first *m* (*m* = 1, 2, 3…20) PLSCs are used to construct a model, and the optimised *m* with largest R^2^ value is used as the effective number of PLSCs (Fig. 1).

To formally check the statistical significance of correlations between the human face and its corresponding personalities, we conducted a permutation procedure. First, for the observed data, we constructed a PLS model using the effective *m* PLSCs, and carried out a LOO step as described above in order to obtain the predicted personality *f*_*i*_ for each individual *i*. As each individual has its true score *u*_*i*_, the correlation ρ can be calculated between ***f*** and ***u***. Second, we randomly permutated the personality scores among different individuals. Based on the permutated sample sets, we repeated the above pipeline to determine the correlation ρ^*^ under the null hypothesis of no association. The permutation procedure is repeated by 1000 times to give rise to a null distribution Π of ρ^*^. An empirical p-value can be calculated for ρ in terms of its ranking position in Π.

Given the effective number of PLSCs in the optimal model, we established a single linear model CPLSC that combines all the effective PLSCs and described how a facial expression changes along the BF scores. Given the effective number of PLSCs *m* and each effective *PLSC*_*i*_ = (*l*_*i1*_, *l*_*i2*_, …, *l*_*ip*_)^T^ *i* = 1, 2, …, *m*, where *p* is the dimension of all the facial image data, we first normalised the *PLSC*_*i*_ as *NPLSC*_*i*_ = *PLSC*_*i*_/||*PLSC*_*i*_||, where $\Vert PLS{C}_{i}\Vert =\sqrt{PLS{C}_{i}^{T}PLS{C}_{i}}$. Then we calculated the standard deviation *sd*_*i*_ *i* = 1, 2, …, *m*, of the sample for each *NPLSC*_*i*_ *i* = 1, 2, …, *m*. Finally, the CPLSC model is calculated as follows:

4 $$CPLSC=\sum _{i=1}^{m}s{d}_{i}\cdot NPLS{C}_{i}$$

### Principal Component Analysis

PCA was carried out by “princomp” in MATLAB. A similar LOO procedure was used to evaluate the statistical significance of association. Briefly, we used all but one samples to train the linear-regression model, and this model was used to predict the excluded sample. The training-testing procedures were implemented on every individual. The predicted and the real data were then compared.

### Partial Correlation Test

Partial correlation test was carried out by “pcor” in R package ppcor^47^. Pearson partial correlation coefficient was used.

### Code Availability

To implement the visualisation and the animation based on the “rgl” package, which is a 3D graphic utility software in R, we developed an open source R package “3DFace”. “3DFace” provides several functions to read the 3D image data, plots the 3D face in different styles and with different gradient colors, and generates fantastic 3D animation for the extracted facial feature. The source code can be downloaded from the website Github, Inc. (https://github.com/fuopen/3dface). The codes used for analysing the reanalysis data are available upon request from the authors.

## Electronic supplementary material

Supplementary Video

Supplementary information
