# Supplementary material for: Signatures of personality on dense 3D facial images
Source: Sci Rep. 2017 Mar 6;7:73. doi: 10.1038/s41598-017-00071-5 (PMC5428336; doi:10.1038/s41598-017-00071-5)
Supplement: Supplementary file 2 — Supplementary information [file 41598_2017_71_MOESM2_ESM.pdf]

Signatures of personality on dense 3D facial images

Sile Hu*[1], Jieyi Xiong*[1,2], Pengcheng Fu[3], Lu Qiao[1], Jingze Tan[4], Li Jin[4], Kun Tang[1]

Supplementary Information.

Supplementary Tables.

Gender	Male					Female				
Personality	E	A	C	N	O	E	A	C	N	O
Pearson correlation with age	-0.139	-0.018	0.17	-0.045	-0.065	-0.064	0.054	0.116	-0.072	-0.011
P-value	0.0049**	0.725	0.00058***	0.371	0.192	0.185	0.264	0.016*	0.137	0.82

Supplementary Table 1. Pearson correlations between personalities and age. Significance: *: p≤0.05; **: p≤0.01; ***: p≤0.001.

Gender	Male	Male	Female	Male	Male	Male	Male	Male	Female
Facial feature	PLS1-3	PLS1-3	PLS1-2	PC3	PC5	PC15	PC16	PC20	PC4
Associated personality	A	C	E	E	A	A	E	C	E
Pearson correlation with age	0.055	0.068	-0.034	0.031	-0.174	-0.026	0.101	0.065	-0.020
P-value	0.134	0.085	0.242	0.529	0.00044***	0.608	0.041*	0.191	0.679

Supplementary Table 2. Pearson correlations between personality-associated facial features and age. Significance: *: p≤0.05; **: p≤0.01; ***: p≤0.001.

Supplementary Figures.

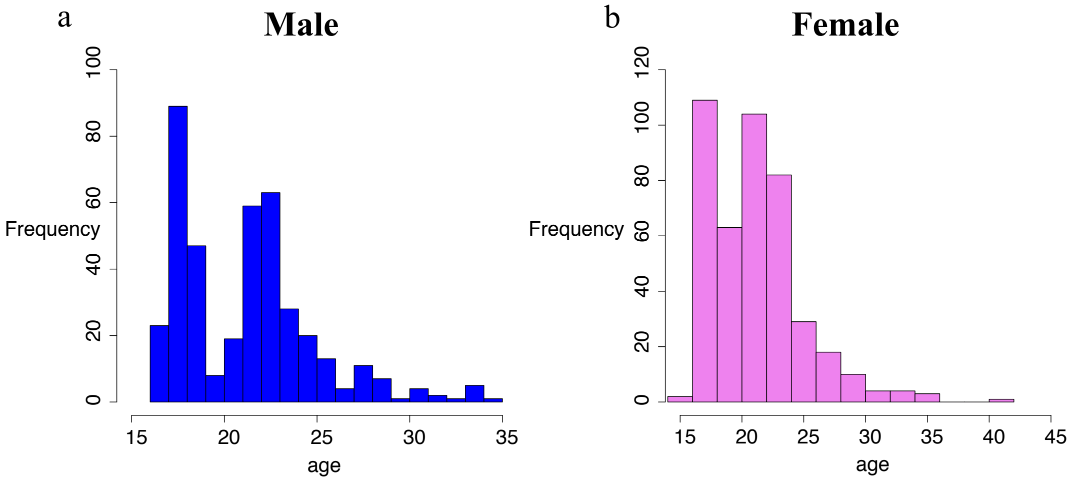

Supplementary Figure 1. The age distributions of male and female volunteers. (a). Age distribution for male. (b). Age distribution for female.

Supplementary Video Legends.

Supplementary video. Continuous transformation of personalities extracted by CPLSC: Agreeableness and Conscientiousness in male and Extraversion in female. The texture images mapped to the surface of 3D animated faces were generated from the mean face of all the samples with respect to their gender separately.
